# Comparing the effects of asynchronous herbivores on New Zealand montane vegetation communities

**DOI:** 10.1371/journal.pone.0214959

**Published:** 2019-04-04

**Authors:** Jamie R. Wood, Janet M. Wilmshurst

**Affiliations:** 1 Manaaki Whenua Landcare Research, Lincoln, New Zealand; 2 School of Environment, The University of Auckland, Auckland, New Zealand; Museu de Ciències Naturals de Granollers, SPAIN

## Abstract

Large herbivores facilitate a range of important ecological processes yet globally have experienced high rates of decline and extinction over the past 50,000 years. To some extent this lost function may be replaced through the introduction of ecological surrogate taxa, either by active management or via historic introductions. However, comparing the ecological effects of herbivores that existed in the same location, but at different times, can be a challenging proposition. Here we provide an example from New Zealand that demonstrates an approach for making such comparisons. In New Zealand it has been suggested that post-19^th^ Century mammal introductions (e.g. deer and hare) may have filled ecological niches left vacant after the 15^th^ Century AD extinction of large avian herbivores (moa). We quantified pollen assemblages from fecal samples deposited by these two asynchronous herbivore communities to see whether they were comparable. The fecal samples were collected at the same location, and in a native-dominated vegetation community that has experience little anthropogenic disturbance and their contents reflect both the local habitat and diet preferences of the depositing herbivore. The results reveal that the current forest understory is relatively sparse and species depauperate compared to the prehistoric state, indicating that deer and moa had quite different impacts on the local vegetation community. The study provides an example of how combining coprolite and fecal analyses of prehistoric and modern herbivores may clarify the degree of ecological overlap between asynchronous herbivore communities and provide insights into the extent of ecological surrogacy provided by introduced taxa.

## Introduction

Large herbivores facilitate a range of important ecological processes and are commonly keystone species within terrestrial ecosystems [1,2]. However, large herbivore communities around the world have experienced high rates of extinction because of severe climate change and human hunting during the late Quaternary period [3–5]. The extinction of large herbivores over the past 50,000 years has affected the structure, functioning and composition of vegetation communities globally, through the disruption of dispersal, disturbance and nutrient cycling processes [6–10]. This process is ongoing. With around 60% of the world’s large herbivore species currently under threat of extinction [11] the complete consequences for terrestrial ecosystems have yet to be fully realized.

Of the practical techniques that have been explored for restoring the ecological processes once provided by threatened or extinct megafauna, the most widely applied has been the introduction of ecological surrogate taxa [12,13]. While active management is one option for restoring large herbivores to ecosystems [14], it has also been argued that such ecological replacement may have occurred inadvertently, with the widespread introduction of large herbivore species around the world over the past few centuries [15,16]. New Zealand is an example of where this may have occurred. In the avian-dominated vertebrate fauna of New Zealand, nine species of the ratite moa (Aves: Dinornithiformes) were the largest (ca. 20–250 kg) native herbivores in terrestrial ecosystems but were rapidly exterminated following initial human settlement some 750 years ago [17]. For ca. 400 years after the extinction of moa, New Zealand had no large herbivores. However, large herbivore communities were once again restored following the introduction of browsing ungulates, particularly deer (Cervidae), during the 19th Century.

While clearly very different organisms, it has been suggested that in terms of their effect on vegetation communities deer may act as ecological surrogates for the extinct moa, and that vegetation communities browsed by deer may be more similar to their natural (i.e. pre-human) state than to the communities that existed during the intervening ‘herbivore-gap’ period [18,19]. In addition, it has been suggested that smaller introduced mammals, such as sheep (*Ovis aries*) and rabbits (*Oryctolagus cuniculus*), may also offer some degree of surrogacy for certain seed dispersal roles and disturbance effects once provided by moa [20,21]. The idea that mammals are true ecological replacements for moa continues to promote interest in popular media, despite researchers having noted a variety of ways in which the ecosystem effects of mammals likely differed from those of moa; the result of different feeding mechanics (i.e. chewing vs. plucking), population densities, and physical disturbance (e.g. ring-barking and soil distubance by ungulates) [22–25]. Moreover, recent evidence of moa diets derived from coprolite (preserved dung) analyses [26,27], and palynological records of forest composition change after the extinction of moa [24,28], also cast doubt on the similarity of mammals and moa in terms of their ecosystem impacts. However, given the temporal gap between moa and introduced mammal species, and the diversity of processes that have contributed towards modifying New Zealand’s vegetation since human settlement, a direct comparison of the ecosystem impacts of moa and mammals has remained elusive. Attempts to recreate ‘natural’ vegetation communities using ungulate exclosure plots clearly do not achieve this aim, as the absence of any browsing in the plots does not mimic the lost pre-extinction avian browse effects [22]. However, fecal analyses may provide the key to such a comparison.

Here, we provide a direct comparison of moa and mammal feeding ecology in New Zealand forests using fecal pollen analysis. Through examining the palynological content of moa coprolites and mammal dung from a montane forest that has experienced little anthropogenic disturbance we detect similarities and differences in diets and forest composition. We then discuss whether the results are consistent with moa and mammals occupying the same niches and having had comparable effects on the local vegetation communities.

## Methods

### Study site

**Fig 1:**
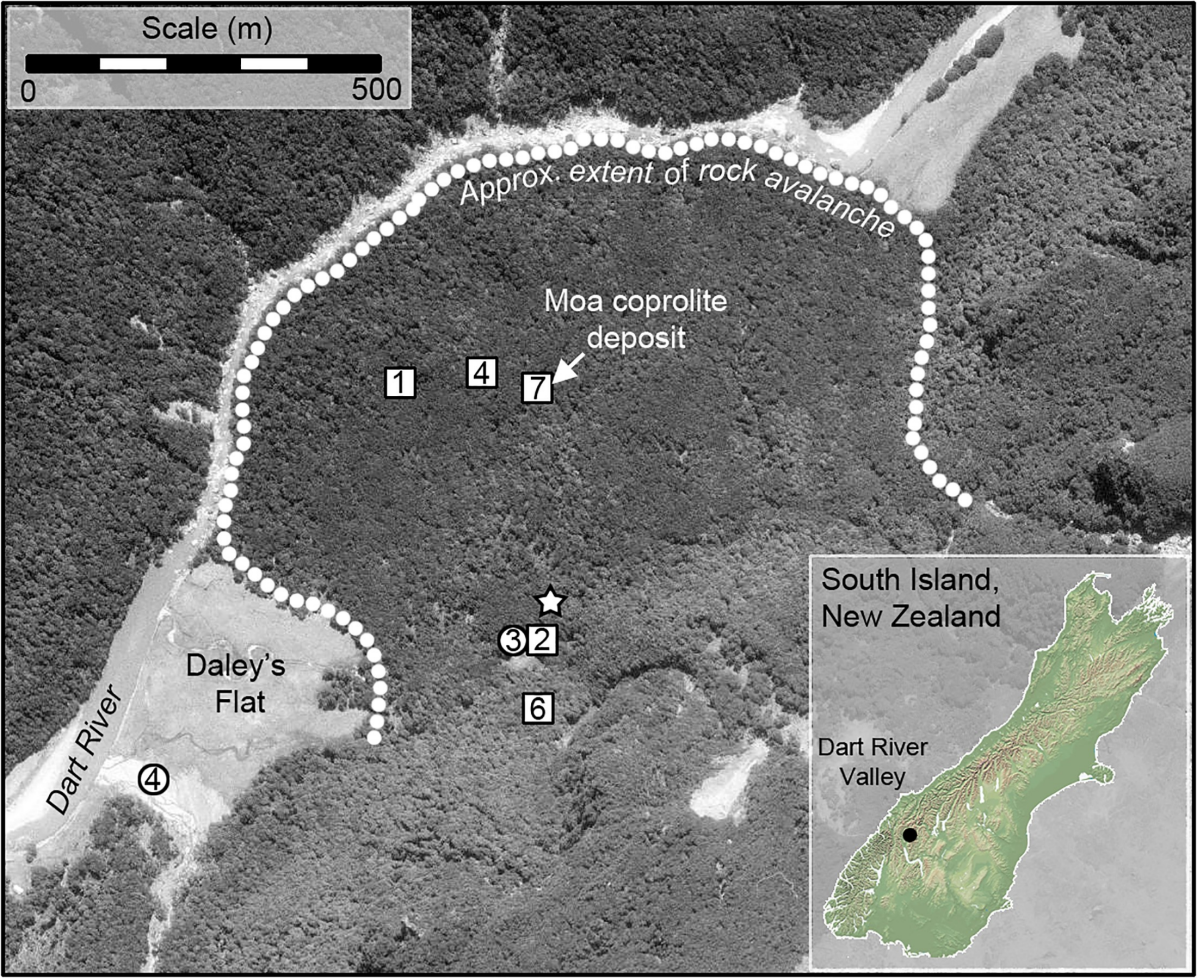
Aerial photograph of Daley’s Flat area, Dart River Valley, New Zealand. The extent of forested rock avalanche deposit and adjacent non-forest habitat are shown, as are the locations and numbers of hare (circles) and deer (squares) pellets collected for this study, and the location of the soil pit from which a pollen stratigraphy was obtained (S2 Fig).

The study site is a large rock avalanche deposit immediately north of Daley’s Flat in the Dart River Valley, West Otago, South Island, New Zealand (Fig 1). The deposit is dated to at least 1,000 years BP [29] and a dry floor beneath an overhanging boulder near the center of the rock avalanche deposit has yielded a significant accumulation of coprolites from four sympatric moa species: South Island giant moa (*Dinornis robustus*), heavy-footed moa (*Pachyornis elephantopus*), upland moa (*Megalapteryx didinus*) and little bush moa (*Anomalopteryx didiformis*) (hereafter referred to by genera alone). The coprolites were deposited over a ~400 year period, ending with the local extinction of moa in the late 14^th^ Century AD [29]. DNA, plant macrofossil and pollen analyses of these coprolites have provided insights into niche partitioning between moa species [27,30]. Here, we use the pollen data presented by Wood et al. [27] to compare against our new pollen data from mammal pellets collected at the same site.

There is no evidence for major prehistoric disturbance of the forest plant community on the Daley’s Flat rock avalanche deposit following its establishment after the earthquake that formed the deposit. The five main prehistoric drivers of major forest change in New Zealand have been fire, climate change, forestry, clearance for agriculture and the introduction of mammalian herbivores. Palynological assessment of soil samples from an 80 cm deep pit excavated on the deposit reveal a successional sequence from ferns through to canopy tree dominance, yet no microscopic charcoal fragments were observed (S1 Fig) suggesting a lack of fire in the local area. This is consistent the lack of post-settlement fire seen in *Fuscospora* forests in higher rainfall areas of the South Island [31]. Multiple partial moa skeletons found lying in-situ on the ground surface within the area of the rock avalanche deposit also support the idea that the native beech (Nothofagaceae) forest on the site has not been burnt since human settlement, as any large fires would have likely destroyed these specimens. With no discernable climate-driven changes in New Zealand vegetation communities over the past 1,000 years, and no forestry undertaken at this particular site, the only remaining potential driver of significant vegetation change at Daley’s Flat is the altered herbivore community.

The forest on the Daley’s Flat rock avalanche deposit is classified as montane red-mountain-silver beech forest [32]. Red beech (*Fuscospora fusca*) and mountain beech (*F*. *cliffortioides*) are the dominant canopy species with sparser silver beech (*Lophozonia menziesii*) in the subcanopy [32]. The sparse small tree, shrub and herb layers within the forest were specifically noted by Mark [32], as was a distinct browse line caused by hares (*Lepus europaeus*) and deer (red deer [*Cervus elaphus*] and white-tailed deer [*Odocoileus virginianus*]). Other herbivores currently occurring at the site include goat (*Capra hircus*) and rabbit [33]. Where small trees and shrubs do occur, these include broadleaf (*Griselinia littoralis*), *Raukaua simplex*, marble leaf (*Carpodetus serratus*), lancewood (*Pseudopanax crassifolius*), celery pine (*Phyllocladus alpinus*), several *Coprosma* species, *Neomyrtus pedunculata* and *Myrsine divaricata* [32].

The adjacent non-forest habitat at Daley’s Flat (Fig 1) is categorized as valley grassland [32]. These non-forest habitats are likely maintained by frost, but periodic flooding may also play a role [32]. The grassland at Daley’s Flat was grazed by cattle up until the late 1980s [34], and together with the presence of introduced grass and herb species [32] is relatively modified compared to its prehuman state. The cattle are likely to have used the forest margins for shelter during inclement weather [34] and this is where their impacts would have been concentrated. We found no evidence (i.e. bones and dung in rockshelters) to suggest they penetrated far into the forest interior. This, together with the fact that shrub-sized trees along the forest margin (within 20 m of forest edge) at Daley’s Flat showed significant and rapid recovery within 10 years of removing cattle browsing [34], demonstrate a negligible impact of legacy cattle browse effects on the current forest vegetation community at Daley’s Flat.

### Mammal dung

In July 2010 we collected deer and hare pellets on and around the Daley’s Flat rock avalanche deposit within 900 meters of the rockshelter where the moa coprolites had been collected [27] (Fig 1). Twenty deer pellets and seven hare pellets were selected for pollen analysis. As moa fed across a range of habitats in the area [27], the mammal pellets were collected from a range of discrete collection locations reflecting these habitat types (Table 1). Collections were made under the Landcare Research Global Concession (CA-31615-OTH) for low impact scientific study on land administered by the Department of Conservation.

**Table 1:** Details of collection localities for deer and hare pellets, Dart River Valley, July 2010.

Locality code	Lat. Long.	Hare n	Deer n	Site description
X10/9	44° 32' 48.86" S, 168° 22' 37.92" E	4		Along a small creek flowing across Daleys Flat, disturbed gravelly ground with vegetation dominated by *Hebe*, regenerating beech (*Fuscospora*), *Coprosma*, *Coriaria sarmentosa*, *C*. *plumosa*, *Acaena* and *Raoulia*
X10/10	44° 32' 46.23"S, 168° 23' 4.35"E		6	A rockshelter 100m from a small clearing beside the old walking track, within beech forest
X10/11	44° 32' 30.27" S, 168° 23' 2.98" E		4	100 m west of the main moa coprolite rockshelter, within beech forest
X10/12	44° 32' 31.2" S, 168° 23' 6" E		7	The main moa coprolite rockshelter, around corner on the uphill side of the rock from the moa deposit, within beech forest
X10/13	44° 32' 44.42" S, 168° 23' 4.77" E	3	2	Fresh dung pellets were collected around the margins of a small grassy clearing within the beech forest
X10/14	44° 32' 30.84"S, 168° 22' 56.21"E		1	Between the main moa coprolite shelter (X10/12) and the walking track, on mossy forest floor beneath beech forest canopy

Mammal pellets were processed for pollen and spore analysis following the same methods used to process moa coprolites from the site. This included removal of the outer surface of pellets, followed by heating in potassium hydroxide for 10 min, treatment with hydrochloric acid, flotation separation of pollen grains using lithium polytungstate (specific gravity, 2.2), acetolysis, staining, and mounting on microscope slides [27]. A minimum of 240 pollen grains were counted, except for five samples where low pollen density prevented this target from being reached (1 deer, *n* = 134 and 4 hare, *n* = 185, 95, 48, 26).

### Statistical analyses

Multidimensional scaling (MDS) analysis was performed using the vegan package version 2.4–2 in R version 3.3.2., to explore the degree of ordihull overlap between fecal samples from different taxa when plotted on the two main axes. Pollen diagrams were plotted using C2 version 1.7.6 [35].

## Results

### Richness of pollen assemblages

**Fig 2:**
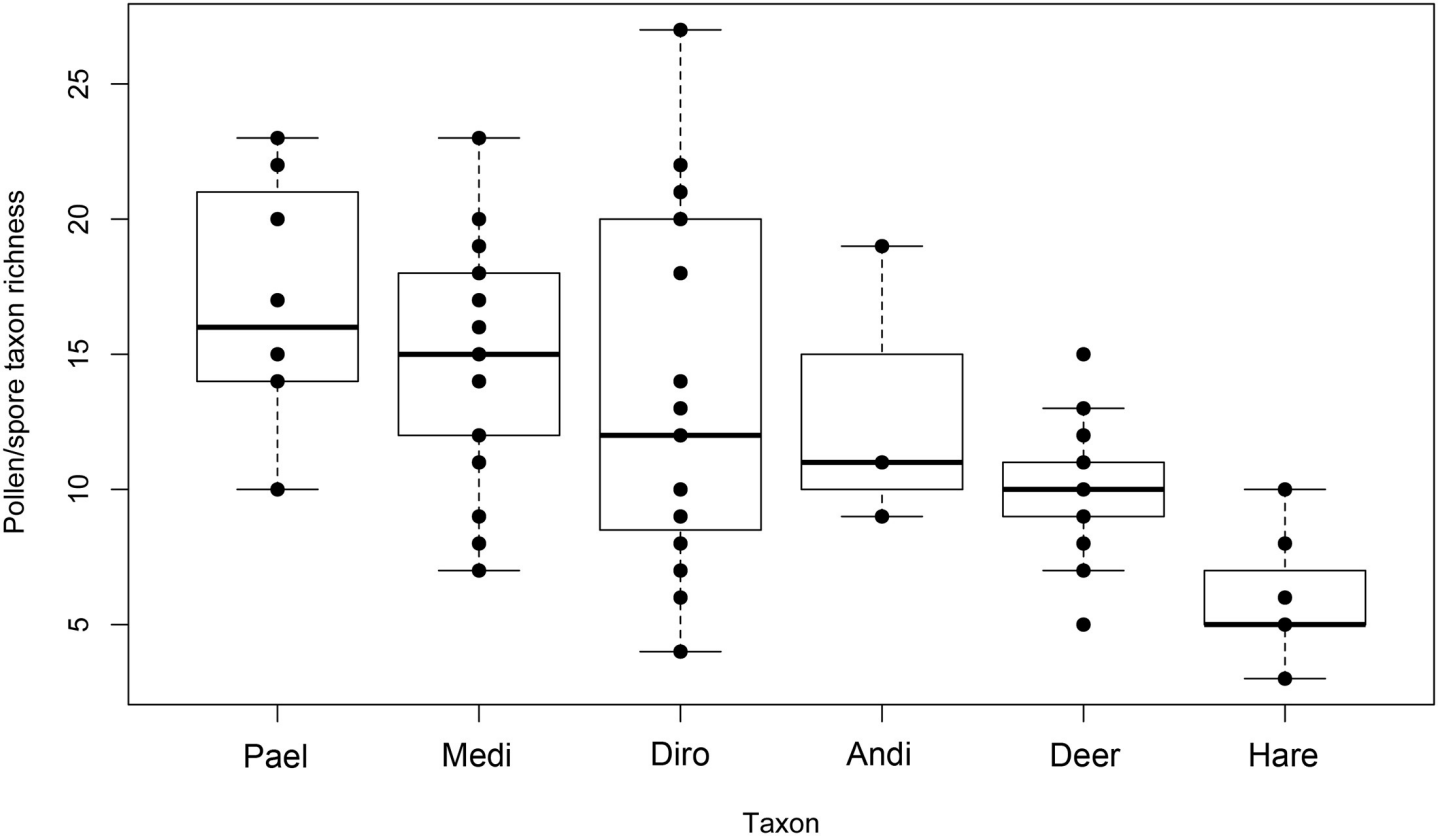
Richness of plant pollen and spore assemblages. Moa coprolites (Pael, *Pachyornis elephantopus*; Medi, *Megalapteryx didinus*; Diro, *Dinornis robustus*; Andi, *Anomalopteryx didiformis*) and deer and hare pellets from Daley’s Flat rock avalanche deposit.

**Fig 3:**
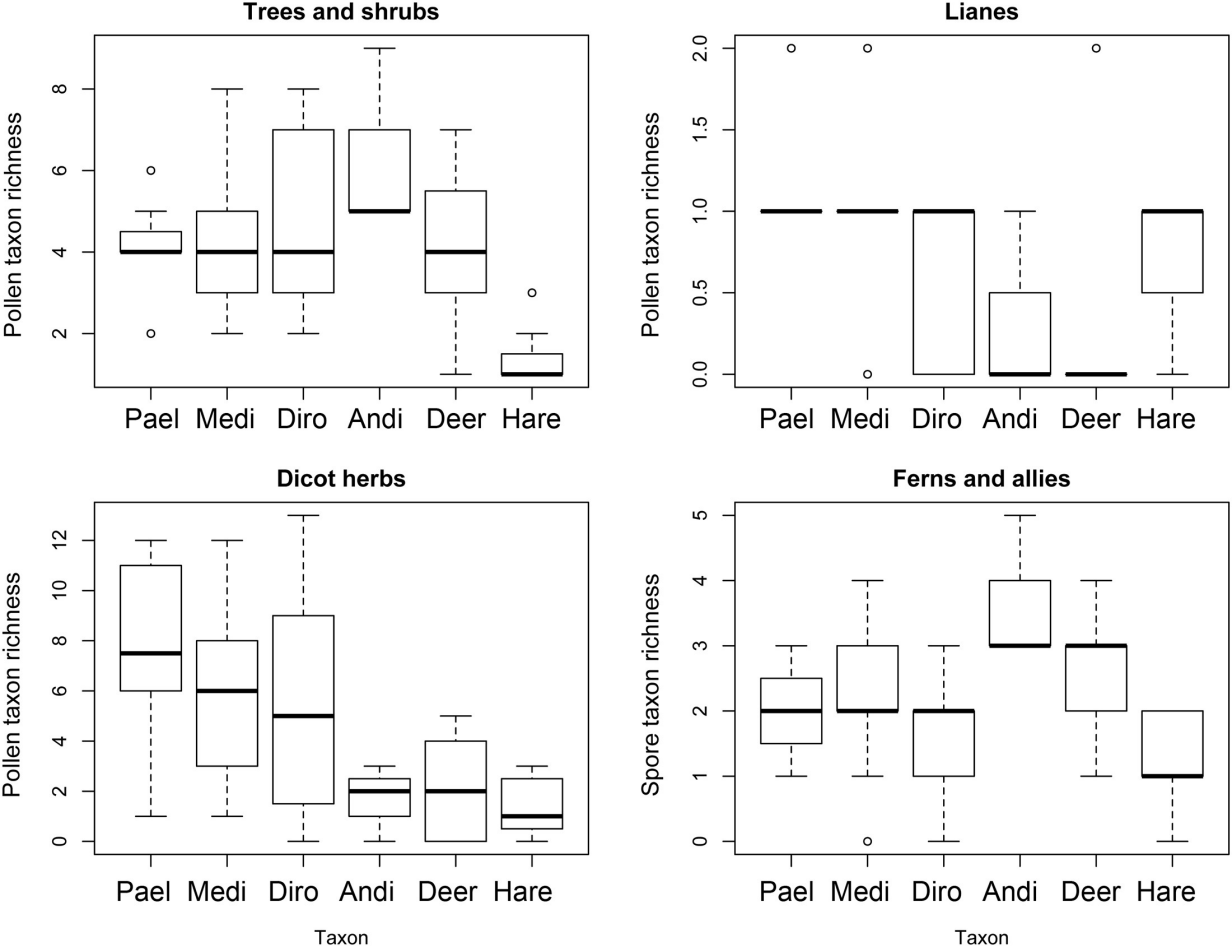
Richness of pollen and spore assemblages attributed to different plant groups. Moa coprolites (Pael, *Pachyornis elephantopus*; Medi, *Megalapteryx didinus*; Diro, *Dinornis robustus*; Andi, *Anomalopteryx didiformis*) and deer and hare pellets from Daley’s Flat rock avalanche deposit.

The overall mean pollen/spore richness of mammal pellets was lower than that of moa coprolites (Fig 2). Coprolites of the forest dwelling *Anomalopteryx* exhibited the greatest mean richness of tree and shrub pollen taxa recorded (mean = 6.33 ± 1.33), while the other moa species were similar to deer (*Pachyornis* mean = 4.12 ± 0.4; *Megalapteryx* mean = 4.43 ± 0.39; *Dinornis* mean = 5.0 ± 0.47; deer mean = 4.25 ± 0.4). Hare had the lowest richness of tree and shrub pollen types per pellet (mean = 1.43 ± 0.3) (Fig 3). *Pachyornis*, *Megalapteryx* and *Dinornis* had the greatest mean richness of herbaceous dicot pollen types in their coprolites (means of 7.75 ± 1.32, 5.71 ± 0.78 and 5.26 ± 0.96 respectively), with coprolites of *Anomalopteryx* and pellets of deer and hare all containing much lower taxon richness (means of 1.67 ± 0.88, 2.2 ± 0.44 and 1.43 ± 0.48 respectively) (Fig 3). In terms of ferns and fern allies, *Anomalopteryx* coprolites (mean = 3.67 ± 0.67) and deer pellets (mean = 2.45 ± 0.21) had higher mean spore richness than coprolites of the other moa species and hare pellets (Fig 3).

### Composition of pollen assemblages

**Fig 4:**
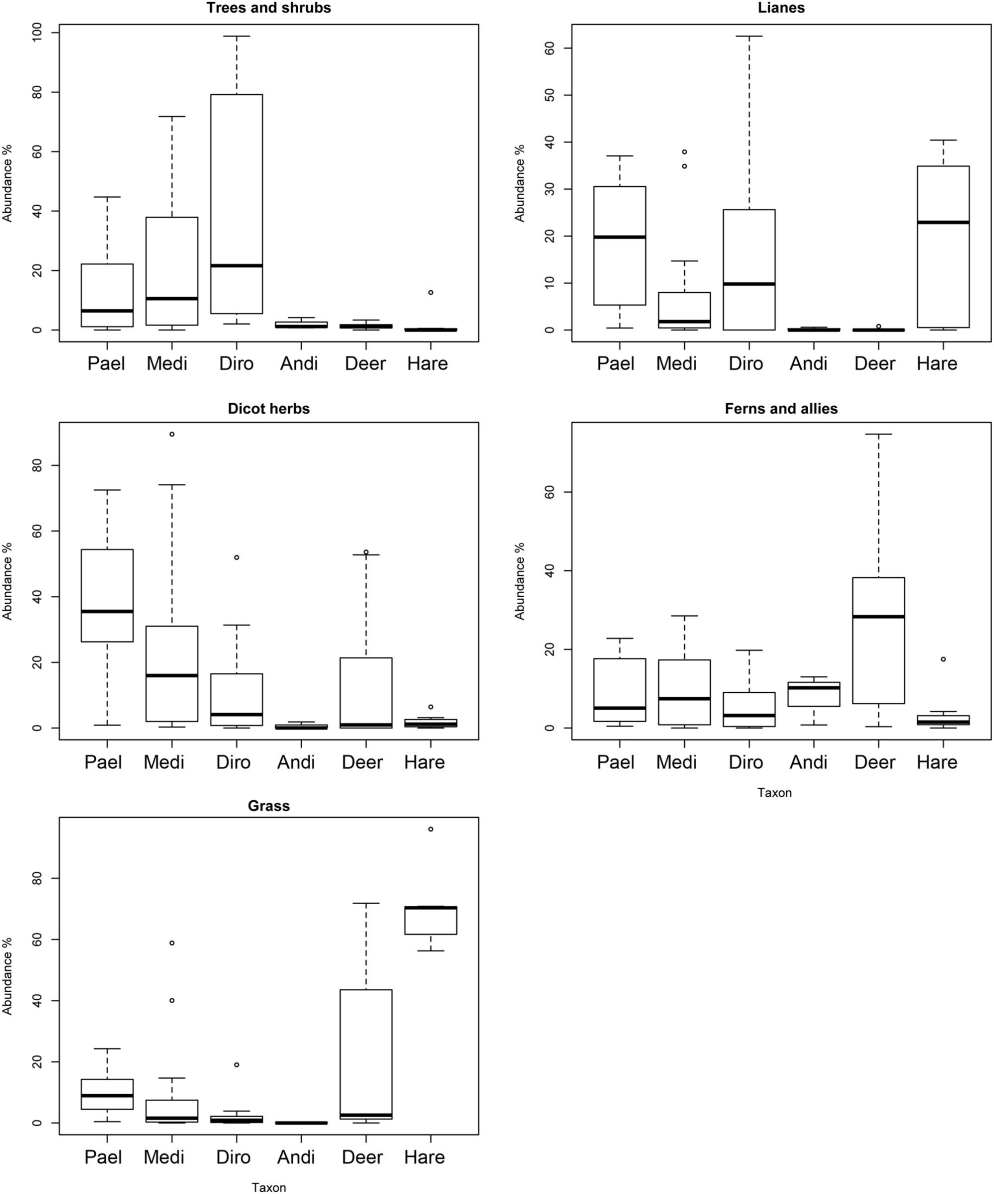
Relative abundance of pollen and spore assemblages attributed to different plant groups. Moa coprolites (Pael, *Pachyornis elephantopus*; Medi, *Megalapteryx didinus*; Diro, *Dinornis robustus*; Andi, *Anomalopteryx didiformis*) and deer and hare pellets from Daley’s Flat rock avalanche deposit. *Fuscospora* pollen is excluded due to its tendency to be over-represented in samples.

**Fig 5:**
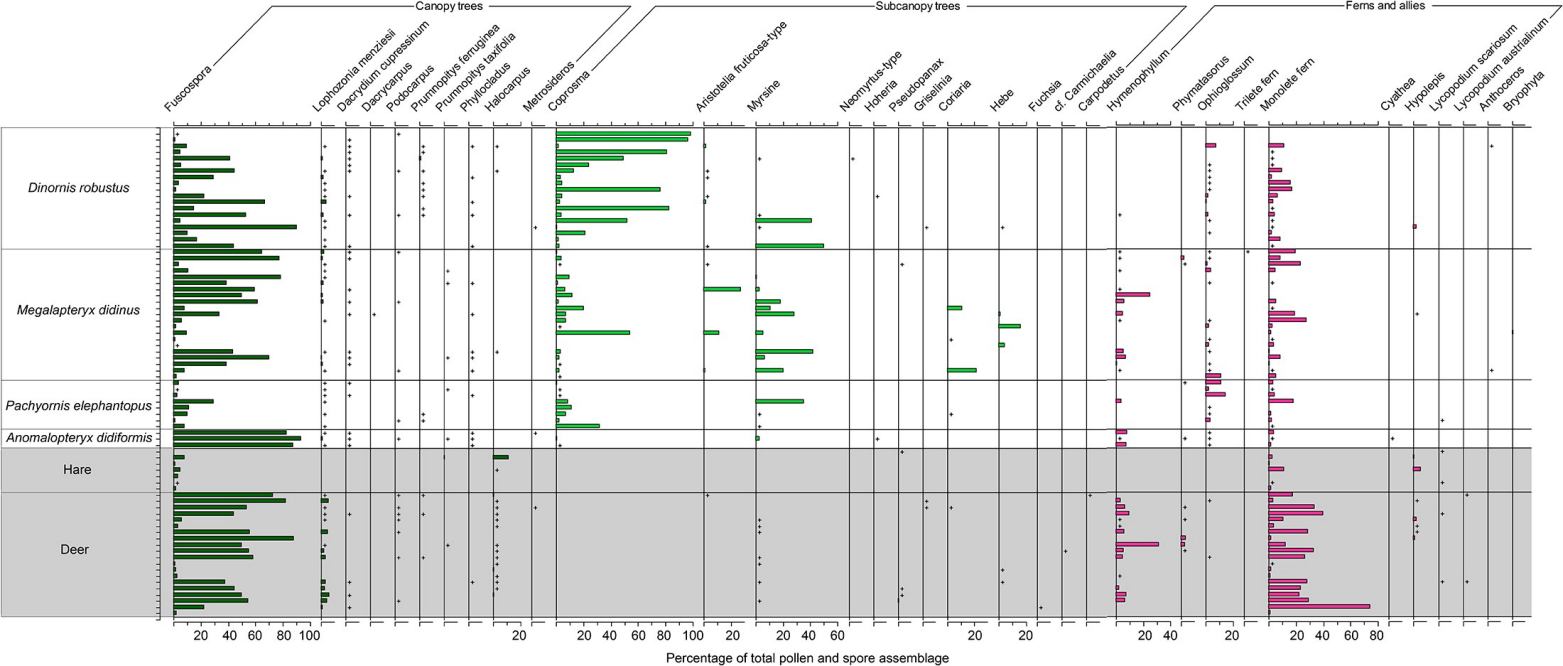
Pollen diagram showing tree pollen and fern spores. Moa coprolites and mammal pellets from Daley’s Flat rock avalanche deposit. Rare pollen types (< 1% total pollen assemblage) are represented by + symbols.

With *Fuscospora* pollen excluded (due to its tendency to be over-represented in samples relative to its contribution to diets; see [26]), tree and shrub pollen represented higher proportions of the pollen assemblages of coprolites from *Pachyornis*, *Megalapteryx* and *Dinornis* compared to *Anomalopteryx* and mammals (where *Fuscospora* was the dominant tree pollen type) (Fig 4). Overall there appeared to be little difference in the occurrence of canopy tree pollen types within moa coprolites and deer pellets (Fig 5). One minor difference was the slightly higher abundances of silver beech (*Lophozonia menziesii*) pollen in deer pellets. As expected, pollen of canopy tree taxa was relatively rare in the hare pellets (Fig 5). The representation of subcanopy trees was quite different between pollen assemblages from moa coprolites and mammal pellets (Fig 5). While subcanopy tree pollen types often occurred in relatively high abundances (mean = 23.76% ± 4.16) in moa coprolites (at least in those species that fed in forest, i.e. *Dinornis*, *Megalapteryx* and *Anomalopteryx*) they were much rarer in deer and hare pellets, and no subcanopy tree pollen type ever exceeded 1% of the total pollen assemblage in any mammal pellets (Fig 5). For example, *Coprosma* pollen showed perhaps the most striking difference, as it was present in every moa coprolite (and representing up to 98.4% of the pollen assemblage per coprolite) but was not recorded in any of the mammal pellets. Other subcanopy tree pollen types such as *Aristotelia*, *Myrsine*, *Coriaria* and *Hebe* occurred at relatively high proportions (>5%) in some moa coprolites but were rare or absent in deer pellets (Fig 5).

Fern spores occurred in almost all samples (Fig 5). Notable differences were the higher frequency of occurrence of *Ophioglossum* spores in moa coprolites (64.7% of coprolites) compared to hare (0%) and deer (10%) pellets (Fig 5). Monolete fern spores (which include common ground ferns such as *Blechnum* and *Polystichum*) generally occurred at higher abundances in deer pellets (mean = 20.03% ± 4.13) compared to moa coprolites (mean = 5.26% ± 0.98), and the overall mean abundance of fern spores was highest in deer pellets (mean = 25.34 ± 4.55) and lowest in hare pellets (mean = 3.68% ± 2.37) (Fig 4).

**Fig 6:**
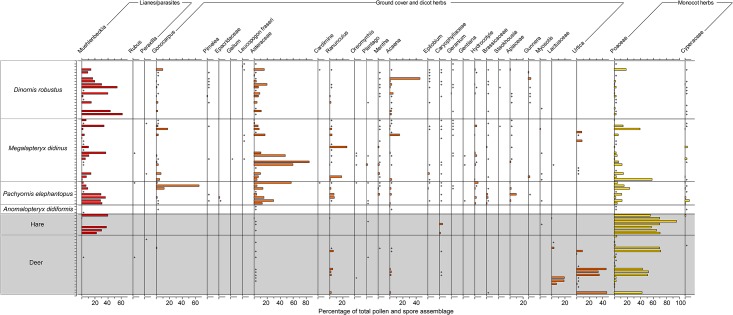
Pollen diagram showing lianes, parasite, ground cover and herbaceous pollen types. Moa coprolites and mammal pellets from Daley’s Flat rock avalanche deposit. Rare pollen types (< 1% total pollen assemblage) are represented by + symbols.

Lianes, and specifically *Muehlenbeckia*, also demonstrated major differences in relative abundance between moa coprolites and mammal pellets (Fig 4). *Muehlenbeckia* pollen differed markedly between moa coprolites and deer pellets (Fig 6) both in terms of occurrence frequency (78.4% of coprolites vs. 5% of deer pellets) and mean abundance (11.1% in coprolites vs. 0.38% in deer pellets). *Muehlenbeckia* pollen was more common in hare pellets than deer pellets and more similar to moa coprolites (Fig 6), being present in five of the seven samples with a mean abundance of 19.1%.

There were also differences in the overall abundance of herbaceous dicots and other ground cover taxa between moa coprolites and mammal pellets (Fig 4). Two taxa, *Gonocarpus* and Asteraceae (the latter of which likely includes mainly herbs but may also include *Olearia* shrubs), were generally at higher abundances in moa coprolites compared to mammal pellets (Fig 6). A range of other ground cover or herbaceous pollen types were detected in moa coprolites but were absent from mammal pellets, including *Pimelea*, Epacridaceae, *Galium*, *Leucopogon fraseri*, *Cardamine*, *Epilobium*, *Geranium*, *Gentiana*, *Hydrocotyle*, *Stackhousia*, Apiaceae, *Gunnera* and *Myosotis* (Fig 6). Herbaceous Lactuceae and *Urtica*, in addition to monocots all occurred at overall higher abundances in mammal pellets compared with moa coprolites (Fig 4). Two types of herbaceous monocot pollen were recorded, including Cyperaceae, which was relatively frequently detected in moa coprolites but only in one hare and one deer pellet (Fig 6); and Poaceae, which was generally more abundant in hare and deer pellets than moa coprolites (Fig 6).

**Fig 7:**
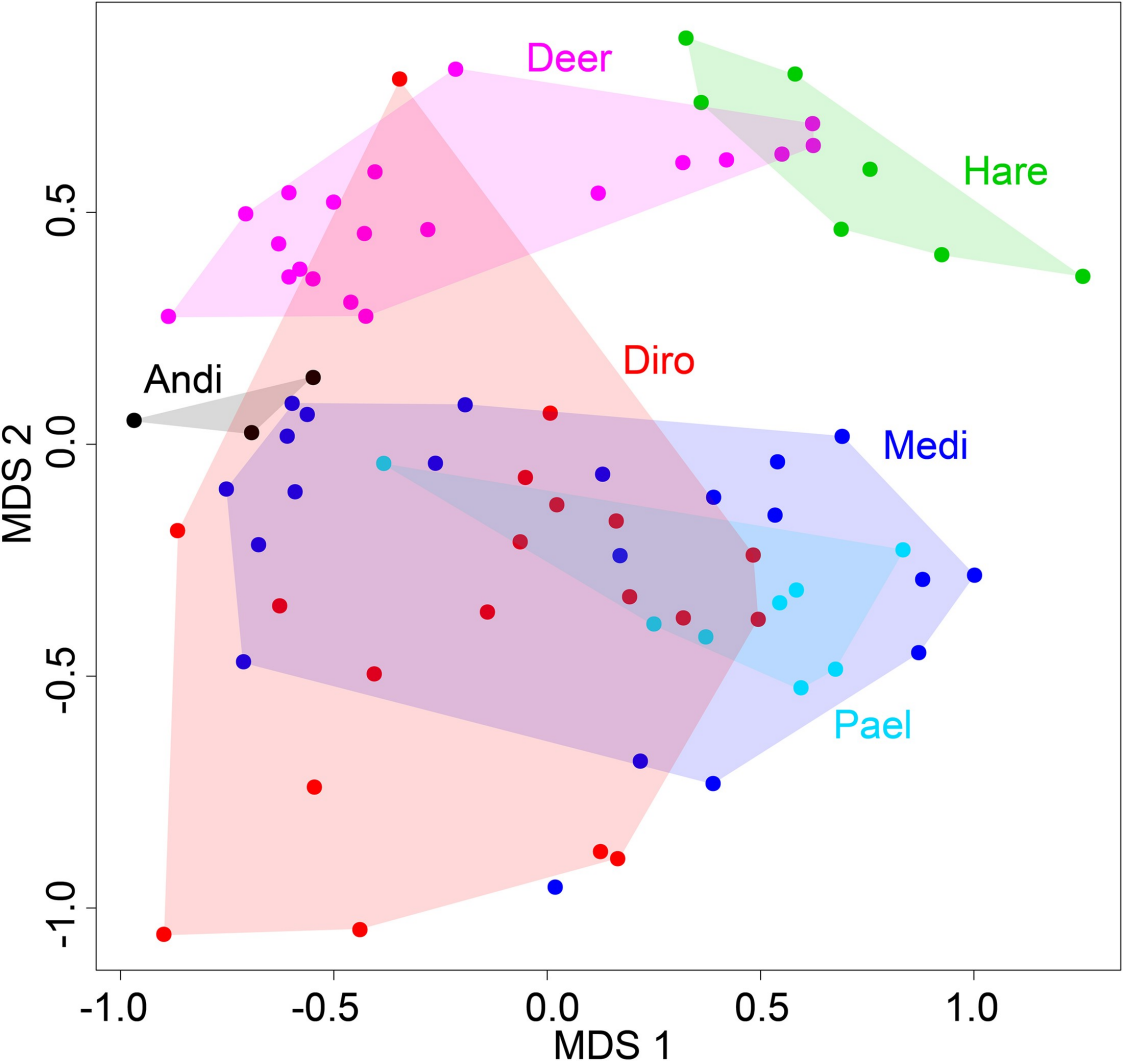
MDS based on pollen and spore assemblages. Moa coprolites (Pael, *Pachyornis elephantopus*; Medi, *Megalapteryx didinus*; Diro, *Dinornis robustus*; Andi, *Anomalopteryx didiformis*) and deer and hare pellets from Daley’s Flat rock avalanche deposit.

The overall composition of pollen and spore assemblages of moa coprolites differed markedly from those of mammal pellets. When the two major MDS axes were plotted, there was no overlap between the hulls of three moa taxa (*Pachyornis*, *Megalapteryx* and *Anomalopteryx*) with those of deer and hare (Fig 7). Just one coprolite of *Dinornis* plotted near to the deer cluster, otherwise all other coprolites of this moa species were also quite distinct from the mammal pellets (Fig 7). The ordihulls of deer and hare also exhibited little overlap (Fig 7) reflecting the different diets and feeding habitats of these two mammal taxa.

## Discussion

### Non-forest habitat

The lower frequency of occurrence and abundance of the liane *Muehlenbeckia* and herbaceous dicots in pollen assemblages from deer pellets compared with those from moa coprolites were some of the more striking differences between the two large herbivore groups. While some of the plant taxa represented by these pollen types occur within the forest understory, they mainly reflect plants that would have been consumed around the forest margins or in non-forested frost-flat habitats, for example *Muehlenbeckia axillaris* [27]. It is clear that a century of cattle grazing in such habitats has left a significant legacy on the composition of non-forest vegetation communities at Daley’s Flat [34], and while most of the plant taxa identified from the moa coprolites can still be found in the local area, competition from introduced grasses together with grazing mammals have probably resulted in current densities being much reduced compared with prehistoric levels. Therefore, with the complicating factors of cattle grazing and exotic plant species to consider, it may not be prudent to infer too much from our data about the differences between moa and deer ecology in non-forested habitats.

As might be expected, the pollen assemblages of the hare pellets different to those of moa and deer. Being a grazing herbivore, hare pellets were dominated by grass pollen. The tree pollen component of hare pellets consisted mainly of ubiquitous *Fuscospora* pollen and other wind-dispersed pollen types that were likely incidentally ingested. However, the relatively high occurrence frequency and abundance (20–40%) of *Muehlenbeckia* pollen in hare pellets (likely *Muehlenbeckia axillaris*, currently growing on the flats) was an interesting result, as this pollen type was also common and similarly abundant in moa coprolites, but not recorded in deer pellets. This provides evidence of direct feeding by hares on *Muehlenbeckia* plants and further exploration of their potential for eating fruit and acting as seed dispersers for this taxon may be worthwhile.

### Forest habitat

As the forested area of our study site was on a rock avalanche deposit, the potential for forest succession to have biased the comparison between moa and deer needed to be considered. Palynological analysis of a forest soil pit located on the rock avalanche deposit revealed a distinct signal of forest succession, with a transition from early dominance of ferns through to later dominance of canopy trees (S1 Fig). An absence of material suitable for radiocarbon dating meant that the age of the succession could not be constrained, and therefore it was not possible to determine whether there was any temporal overlap with the moa coprolites. However, by plotting the pollen/spore content of 20 radiocarbon dated moa coprolites from the site (S2 Fig) it was possible to show a lack of succession signal, indicating that moa may have only inhabited the site after tall forest had returned.

A key difference between the two large herbivore groups was the low richness and abundance of forest subcanopy tree and shrub pollen types in deer pellets compared with moa coprolites. This difference must be interpreted with the understanding that pollen and spore assemblages from herbivore dung reflects a combination of diet and habitat, and of pollen dispersal. For example, the presence of pollen types that are produced in low abundance and have limited wind dispersal capability (e.g. *Peraxilla*, see [36]) likely demonstrates feeding upon that plant taxa, while pollen types that are produced in high abundance and are readily wind-dispersed (e.g. *Fuscospora*, see [36]) may be ubiquitous in the environment and can also be present due to incidental ingestion [26]. Therefore, pollen traits are important to consider when examining the differences between moa coprolites and mammal pellets presented here. The two most abundant subcanopy tree and shrub pollen types in moa coprolites (*Coprosma* and *Myrsine*) are produced in relatively high abundance and are partly or entirely wind dispersed [36]. Accordingly, while their presence in coprolites and pellets at very high proportions (e.g. >50%) likely reflects consumption, their occurrence may also partly reflect their abundance within the local forest understory. Therefore, the relative rarity of such palatable subcanopy tree and shrub pollen types in deer pellets can be directly interpreted as reflecting a sparser woody component to the forest understory today, compared to that which existed in the presence of moa.

**Fig 8:**
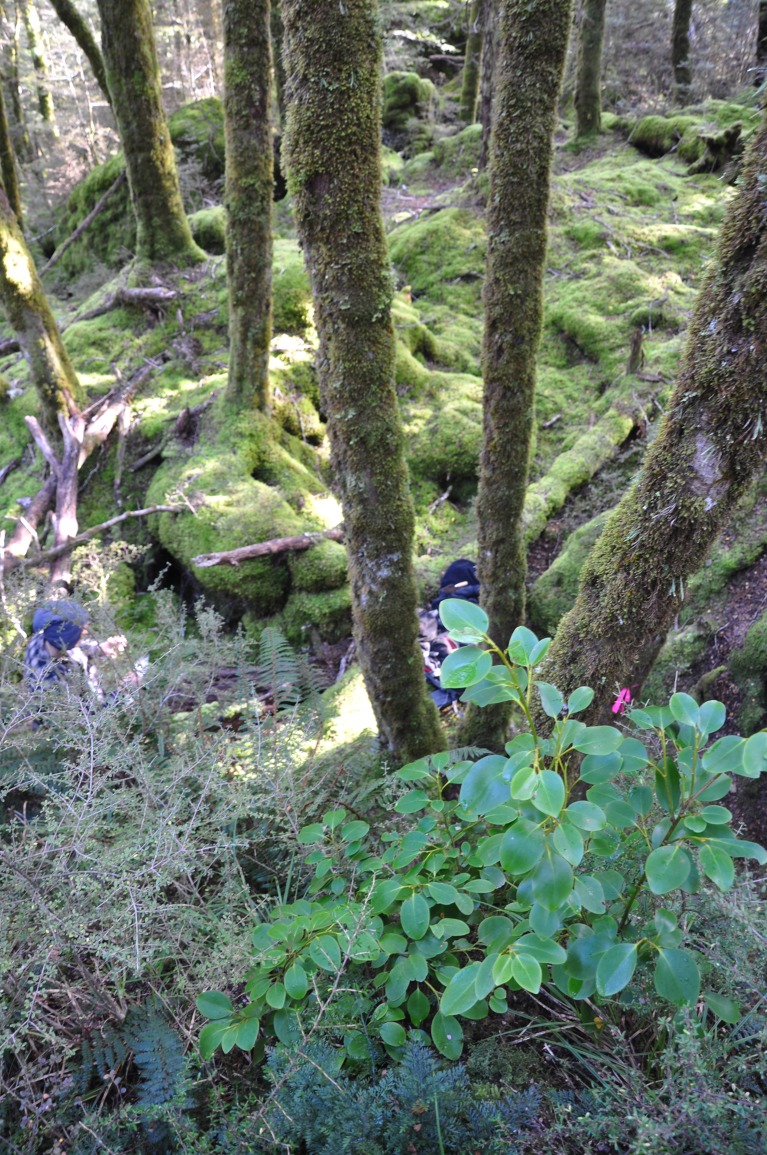
View from top of large boulder inaccessible to deer, Daley’s Flat rock avalanche deposit. A dense and species-rich patch of plants in the foreground (including *Coprosma* spp., *Griselinia*, *Carex*, *Asplenium*, *Hymenophyllum*, *Polystichum* and *Notogrammitis*) contrasts with the sparse forest understorey in the presence of deer in the background (mainly *Fuscospora* trees and mosses).

During fieldwork on the Daley’s Flat rock avalanche deposit we observed that the forest understory was relatively sparse in small trees and shrubs, but that relatively dense and species-rich stands of these existed on top of very large boulders (>5m tall) that are inaccessible to deer (Fig 8). The low abundance of these pollen types in the deer pellets cannot be explained by differential diet preferences, as most of these taxa (*Coprosma*, *Aristotelia*, *Myrsine*, *Hebe*, *Pseudopanax*, *Griselinia* and *Fuchsia*) include preferred food species of deer, or species for which no avoidance has been detected [37]. Therefore, it seems clear that higher population densities and/or greater browse intensity of deer compared to moa have driven the loss of these palatable subcanopy trees and shrubs, which had remained relatively common in the presence of moa.

The higher proportion of monolete fern spores (which are also ubiquitous and do not necessarily reflect direct consumption) in deer pellets compared with moa coprolites reflects a relatively higher abundance of ferns within the current forest understory compared to the past. Similar trends have been reported by herbivore exclosure plot experiments within New Zealand forests, where deer have been shown to drive both a reduction in palatable tree/shrub density and an increase in the density of ferns (particularly *Blechnum* and *Polystichum*) (e.g. [38]). Moreover, a study using pollen/spore analysis of forest soil cores showed that on average monolete fern spores were more abundant following the introduction of deer than prior to moa extinction [28].

Our data on moa coprolites and deer pellets from a forested environment is the first direct comparison of the droppings of these two large herbivore groups in New Zealand at the same location and in a vegetation community that has not experienced clearance or other significant alteration by human activities. Overall our results reflect significant differences in the structure and composition of the forest understory in the presence of deer, compared with the forest understory in which moa once roamed. They indicate that the current forest understory is relatively sparse and species depauperate compared with the prehistoric state, which is consistent with what is known about the impacts of deer browse on New Zealand forest understories. There are a number of reasons why the effects of mammals (such as deer) and moa may have differed substantially, but our data cannot resolve the relative importance of these in driving the differences observed in our study. These could include higher population densities [21], perhaps in part driven by a lack of natural predators (e.g. for deer), a novel feeding apparatus (grinding teeth compared with a beak) for which the New Zealand flora may be maladapted [22], and novel impacts on soil structure [25].

A range of introduced herbivore species are now widespread throughout New Zealand, offering the possibility of comparable studies at locations where moa coprolites have been recovered, for example on the Garibaldi Plateau [26]. As with the Daley’s Flat site, it was also noted that plants which occurred commonly in moa coprolites from the Garibaldi Plateau were now largely restricted to sites inaccessible to deer and hares [26]. While pollen can be driven both by diet and habitat, the incorporation of macrofossil and molecular identification techniques (as used for moa coprolite analyses) will provide more detailed information specifically on diet and seed dispersal and allow these aspects of avian and mammalian herbivore ecology to be compared.

While some vegetation communities appear to respond in a consistent way to herbivory, irrespective of the herbivore species (e.g. [39]), the situation we present here provides a further example of where different large herbivores have different effects on a vegetation community. Although more distantly related herbivores (i.e. mammals and birds) are likely to have greater differences in their ecosystem effects, there are several examples of where a high degree of surrogacy does seem to be provided (e.g. birds and mammals [40]; birds and tortoises [41]). However, fecal analyses have shown that the case of moa and ungulates in New Zealand forests, is not one of these. On a global scale it may be rare to find extinct herbivore coprolites at sites with relatively unmodified vegetation that is broadly comparable to that which existed in the past. However, in cases where such specimens can be found our study has demonstrated the potential for using them to gain deeper insights into the relative impacts of asynchronous herbivores on plant communities.

## Supporting information

S1 Table Raw pollen count data from moa coprolites (from [26]) and deer and hare droppings, Daley’s Flat, Dart River Valley, New Zealand. (CSV)

S1 Fig Pollen diagram for soil pit excavated on rock avalanche deposit, Daley’s Flat, Dart River Valley, New Zealand. (TIF)

S2 Fig Composite pollen diagram, constructed using pollen assemblages from radiocarbon dated moa coprolites, Daley’s Flat, Dart River Valley, New Zealand. (TIF)
