# Supplementary figures and images for: Comparing the effects of asynchronous herbivores on New Zealand montane vegetation communities
Source: PLoS One. 2019 Apr 4;14(4):e0214959. doi: 10.1371/journal.pone.0214959 (PMC6448933; doi:10.1371/journal.pone.0214959)

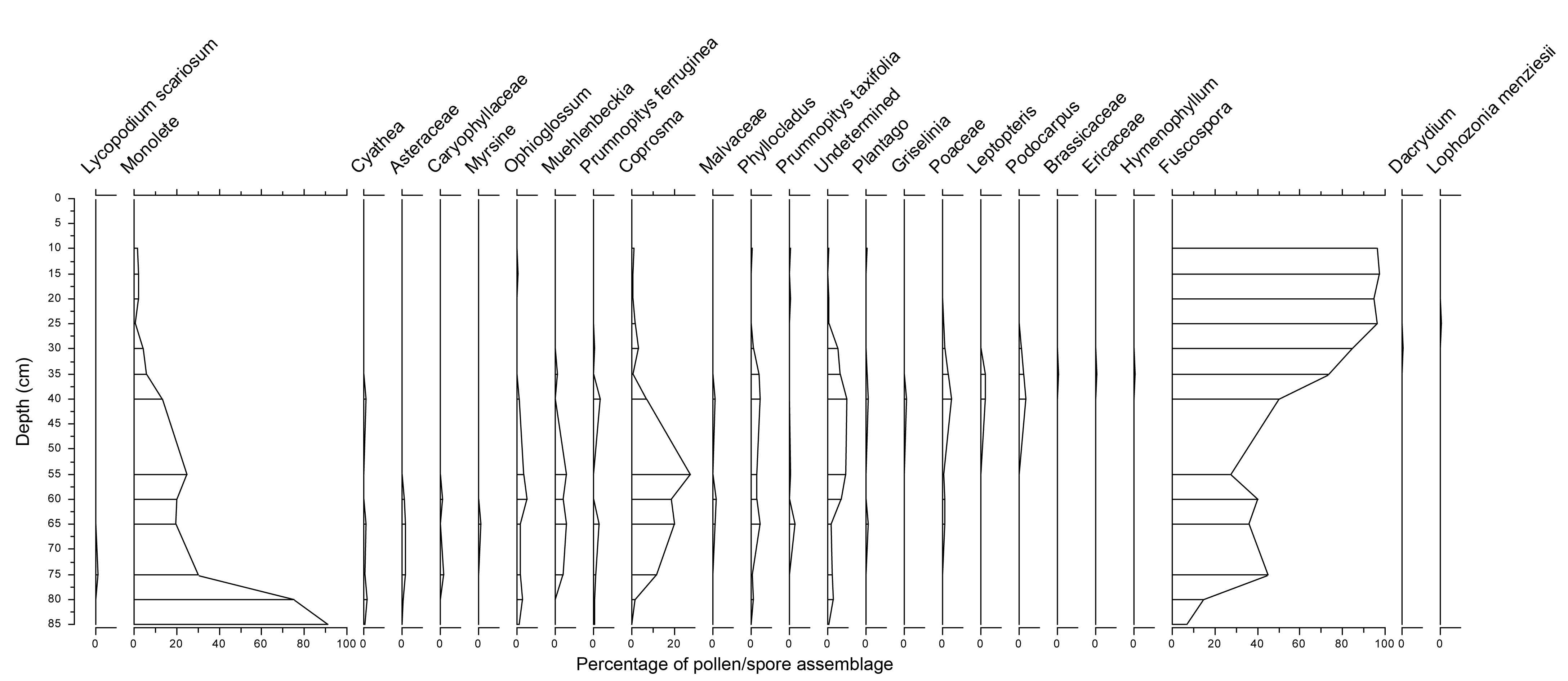

Supplement: S1 Fig — (TIF) [file pone.0214959.s002.tif]

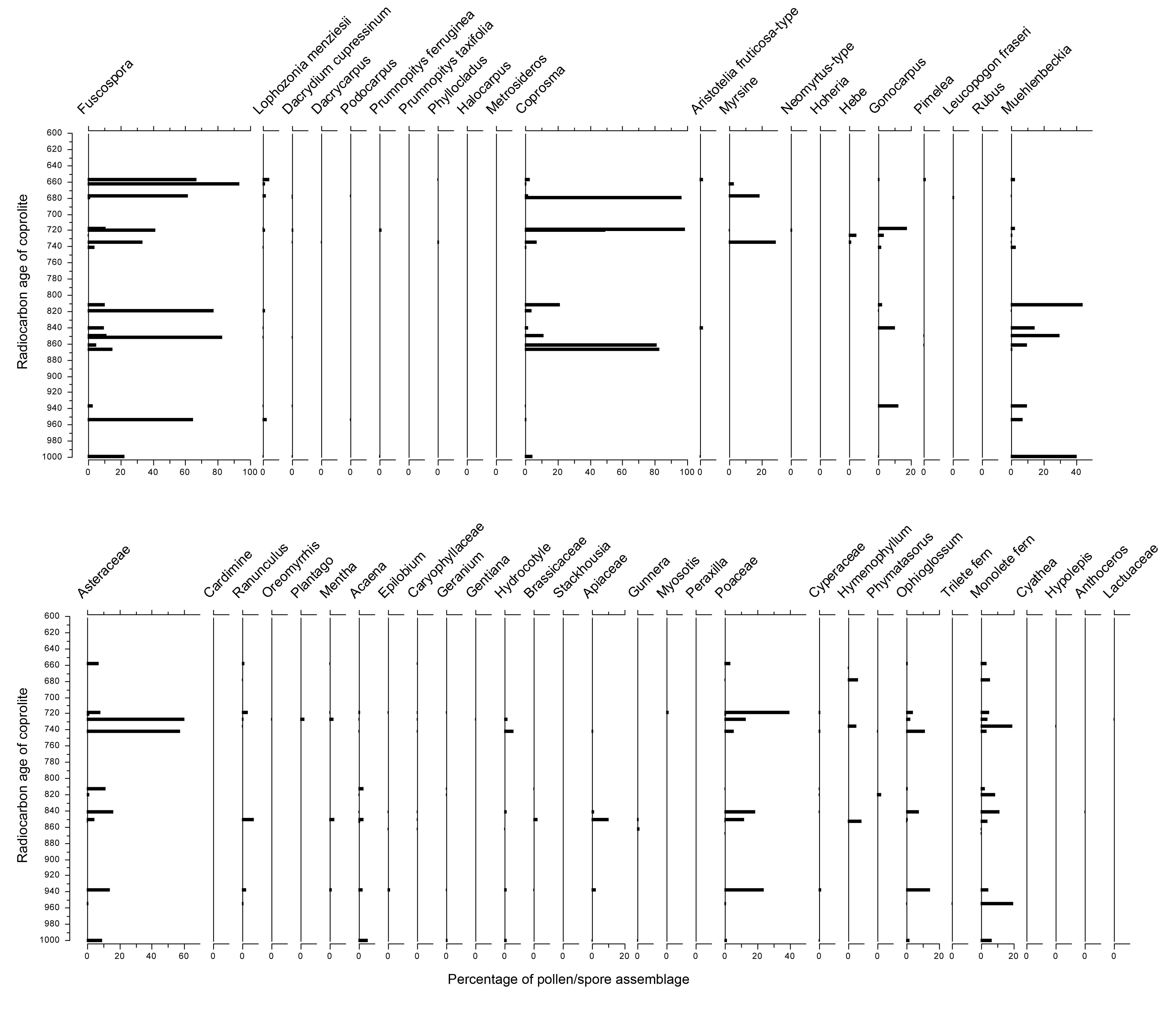

Supplement: S2 Fig — (TIF) [file pone.0214959.s003.tif]
